# Infection Control in Dental Practice during the COVID-19 Pandemic: What Is Changed?

**DOI:** 10.3390/ijerph20053903

**Published:** 2023-02-22

**Authors:** Mario Caggiano, Alfonso Acerra, Stefano Martina, Marzio Galdi, Francesco D’Ambrosio

**Affiliations:** Department of Medicine, Surgery and Dentistry “Scuola Medica Salernitana”, University of Salerno, Via Allende, 84081 Baronissi, Italy

**Keywords:** COVID-19, SARS-CoV-2, dental practice, dentistry, survey, prevention procedures, infection in dentistry

## Abstract

The COVID-19 pandemic has profoundly changed our lives. Since the SARS-CoV-2 was discovered, many studies have been done on the transmission mode, its replication within humans, and its survival even in the outside environment and on inanimate surfaces. Undoubtedly, health care workers have faced the greatest risks because of their close contact with potentially infected patients. Of these, dental health care professionals are certainly among the most vulnerable categories, precisely because infection occurs with the airborne virus. The treatment of patients within the dental office has changed profoundly, respecting all preventive measures towards the patient and the practitioners themselves. The aim of this paper is to understand whether the protocols changed for the prevention of SARS-CoV-2 infection among dentists remained even after the most acute phase of the pandemic. In particular, this study analyzed habits, protocols, preventive measures, and any costs incurred in the COVID-19 era for the prevention of SARS-CoV-2 infection among dental workers and patients.

## 1. Introduction

Since the spread of SARS-CoV-2, research on its modes of transmission, how it survives in the external environment and its length of survival have been carried out, in order to better understand its transmission from person to person [1]. Human-to-human transmission appeared to be primarily through close contact with symptomatic people with COVID-19, and the main way of infection was through respiratory droplets when patients sneezed or coughed [2]. Even though the virus was most contagious when the patient was symptomatic, human-to-human transmission also occurred in patients with mild or no symptoms [3]. Furthermore the virus could survive outside living organisms, in aerosol, or on inanimate materials [4,5].

Due to the pathophysiological characteristics of the COVID-19 syndrome, the particular transmissibility of this virus and the high globalization, the epidemic emergency from China has spread rapidly all over the world, so much so that it was declared a pandemic by World Health Organization (WHO ) on 11 March 2020 [6,7]. 

Undoubtedly, healthcare has undergone the greatest changes, as many healthcare professionals are those in closest contact with patients infected with COVID-19 [8,9]. These operators, in particular dental health care professionals, had to follow strict protocols for the management of sick patients and to prevent the contagion and spread of COVID-19 [10].

In fact, dentists are the frontline, healthcare professionals who have a high risk of contracting COVID-19 because they work closely with the patient, breathing in the aerosols produced by dental procedures, and touch contaminated surfaces [11].

Dentists themselves are aware of the risks they are exposed to during clinical practice and have shown concern about the consequences for the profession [12,13,14]. Therefore it has been necessary to identify a series of specific measures to prevent infection and spread in the dental office [15,16,17].

A literature review conducted by Induri et al. examines preventive measures against COVID-19 implemented in dental offices to protect patients and dental health care practitioners [18]. Guidelines for infection prevention and control within dental offices were described to make the workplace safe, even before the COVID pandemic, precisely because this environment is very susceptible to different types of infection [19,20]. The use of personal protective equipment (PPE) such as gloves, masks, eye protection, and gowns and the use of adequate ventilation and disinfection procedures among patients can greatly reduce the risk of infection [21,22,23,24,25].

The vaccination campaign and preventive measures taken decreased the perception of the dangers and spread of the virus, but the last few months saw the greatest spread of the highly infective omicron variants (BA.4, BA.5), which resulted in a massive increase in infections [21]. In particular, in the three months analyzed for our study (May–July 2022), there was a percentage increase in infections in Italy compared with the previous year of 377%, 1747%, and 33,330%, respectively (https://www.salute.gov.it/portale/home.html, accessed on 19 February 2023).

Based on the above, the aim of the present study is to understand whether habits, protocols, and any costs incurred in the COVID-19 era for the prevention of SARS-CoV-2 infection have remained in place among Italian dentists, even after two years post the end of lockdown in Italy.

## 2. Materials and Methods

### 2.1. Study Design

The present cross-sectional study was conducted by sending an online survey questionnaire to Italian dentists, from May 2022 to July 2022.

The survey was created on an online survey development cloud-based software called SurveyMonkey^®^ (SVMK, San Mateo, CA, USA).

### 2.2. Study Participants

The participants were approached via Facebook, WhatsApp, and mailing list (no paid advertising was used). The participation was voluntary and with no compensation. Participants’ personal data were protected and there were no inhuman questions or investigations. All participants gave permission for the use of their data, which were collected anonymously. No personal health information was collected, so ethical approval was not necessary.

### 2.3. Data Collection

The questionnaire, developed by a group of dentists with different dental specialties, consisted of 37 multiple-choice questions, which were sorted into three main sections.

The first section was about participant personal data such as age, gender, SARS-CoV-2 infection or vaccination, and about professional characteristics of study participants, such as working experience (in years), place of work, and main clinical practice (oral surgery, endodontics, orthodontics, pediatric dentistry, periodontology, prosthetics/implantology, and others) [26]. 

The second section was about the preventive measures implemented during the pandemic era and devices used daily in dental clinical practice to prevent SARS-CoV-2 infection (use of PPE in daily clinical practice and frequency of PPE replacement, and patient management upon arrival at the dental office).

The last section was about the costs incurred for all protective devices and measures, whether these were helpful in preventing infection, and whether the protocols adopted were relaxed after vaccination.

### 2.4. Statistical Analysis

Frequencies and percentages for categorical data were computed. A chi-square test was used to assess the association between gender (male vs. female), age, main activity, COVID-19 infection, and the use of specific PPE and sanitization protocols. A standard statistical software package (SPSS, version 22.0; SPSS IBM, Armonk, NY, USA) was used. The level of significance was set at *p* < 0.05.

## 3. Results

Out of the 392 dentists who participated in the study, 280 (182 M, 98 F) completed the questionnaire. Personal data and participants’ professional characteristics, included in Table 1, showed that most of the participants were men (65%), between 35 and 45 years of age (112/280, 40%), who almost all work in Italy (275/280, 98.2%).

The second section was the most substantial one. It analyzed, as shown in Table 2, the measures implemented and devices used in dental clinical practice for the prevention of SARS-CoV-2 infection [27]. Non-urgent appointments were rescheduled when the patient reported a body temperature above 37.5°C or the presence of symptoms attributable to COVID-19. However, only 56/280 of the interviewed dentists (20%) supposed that they were infected within the dental office. 

In addition, this section assessed the infection prevention measures that the dentists participating in the questionnaire used, such as the type of mask worn, both in the operative and non-operative phase, and its replacement frequency, the use of disposable gowns/suits (154/280, 55%) and their average frequency of replacement. All of these measures contributed to the individual protection of the operator during clinical activity, according to the participants in the questionnaire. Yet these same measures have burdened the costs incurred by clinicians, as shown in Table 3. 

Both regarding SARS-CoV-2 infection in general and within the dental environment, there was no statistically significant difference in relation to sex, age, branches, and the use of PPE, except for the average mask replacement frequency. Indeed, among the dentists who were infected with SARS-CoV-2, 138 (61.6%) changed their masks only once a day (*p* = 0.03).

## 4. Discussion

This cross-sectional web-based survey study aimed to understand the habits, protocols, and any costs incurred during the COVID-19 era for the prevention of SARS-CoV-2 infection among dentists, the dental team, and patients.

This study also aimed to assess whether the preventive measures applied during the COVID-19 pandemic were useful, in the opinion of the respondents, and whether these will be maintained in the future.

### 4.1. Personal Protective Equipment Daily Use in Dental Practice to Prevent SARS-CoV-2 Infection

A total of 95% of respondents declared to have implemented the use of PPE during daily dental practice to prevent SARS-CoV-2 infection.

FFP2 masks, the high filtration valveless respirators, were the preventive device most used by Italian dentists who responded to the study, both during the non-operative phase and during dental procedures, with percentages of 55% and 60%, respectively. These results are consistent with studies showing the increase and effectiveness of mask use to reduce the risk of COVID-19 infection. Regarding the frequency of mask replacement, only 15% of the interviewees followed the protocol introduced by Amato et al. (mask replacement after any clinical procedure), while 29% replaced their mask once a day and 30% every 8 h [6]. As evidenced by the tests conducted by the Institute for Hygiene and Environmental Medicine of the University of Greifswald in Germany on the persistence of coronaviruses on inanimate surfaces and their inactivation with a biocidal agent, the virus remains on the facemask, which is why it must be replaced after the visit [13]. In accordance with this, and not surprisingly, the only condition that resulted in a statistically significant increase in SARS-CoV-2 infection risk among Italian dentists is changing the FFP2 mask only once a day.

Fortunately, 80% have used protective face shields since the contact of the droplets released during dental procedures with the ocular structures can be a possible source of transmission of the virus [14]. The use of this device in 65% of cases was associated with the use of safety glasses, essential in some fields of dentistry [28].

About half of the respondents used disposable gowns and hoods (55% and 45%, respectively). The reduced evidence of their use may be related to the fact that they were considered ineffective in prevention, considering that 30% of dentists who used disposable gowns only replaced them once a day. In contrast, among those who used disposable headgear, their replacement was performed among patients in 73% of cases.

### 4.2. Measures Implemented in Dental Practice to Prevent SARS-CoV-2 Infection

The results of this study are in agreement with other authors who underlined the importance of the anamnesis, which has become of fundamental importance to prevent SARS-CoV-2 contagion in the dental practice [22,29]. The triage should be included in the anamnestic sheet to evaluate any contacts with people suffering from COVID-19 or at risk of exposure [23]. Our study found that the patient’s body temperature check was performed by 100% of the interviewed dentists. In particular, 32.9% of the respondents stated that at least once, the body temperature was higher than 37.5 °C, and when this happened, as suggested by the study of Amato et al., the appointment was postponed by 100% of those who answered “yes” to the question.

In addition, 10% of the dentists interviewed said they had treated SARS-CoV-2 positive patients who needed urgent and non-deferrable treatment, although the literature recommended in these cases to refer them to the hospital, which could adequately treat a patient affected by COVID-19 [6,30].

Another procedure that should not be underestimated is hand hygiene before and after dental interventions with hydroalcoholic solutions or water and soap, which cause the inactivation of the virus [24,25]. This should be considered a mandatory habit even before the COVID-19 pandemic; however, this study found that 7.9% and 3.9% of dentists did not disinfect their hands before and after performing clinical procedures, respectively.

On the other hand, there was no lack of SARS-CoV-2 infection prevention for patients, as in 65% of cases, access to the operating room was allowed only with the use of adequate PPE. In particular, 82.4% of dentists provided their patients with shoe covers, 38.5% with disposable headgears, and 29.7% with disposable gowns [31,32].

The survey showed that dental practice operating rooms were ventilated for at least 15 min in between clinical appointments from 67.9% of the respondents. This practice is essential to ensure that droplets released into the air during procedures can leave the dental office [33]. Indeed, dentists use high- and low-rotation instruments to perform various procedures, including cavity preparations, endodontic accesses, prophylaxis, and oral surgery. Due to the speed of rotation, these tools are used with water jets or saline solutions that produce aerosols [34]. A comparative study published in the New England Journal of Medicine by van Doremalen et al. found that SARS-CoV-2 remained viable in aerosols for up to 3 h with a half-life of 1.5 h [5]. If necessary, the use of the rubber dam is recommended, which minimizes the amount of aerosol coming from the oral cavity [22]. An alternative to opening windows, if such a possibility does not exist, is the use of UV lamps or air filters with HEPA (High-Efficiency Particulate Air) or negative pressure air chambers [15].

Since SARS-CoV-2 survives for several hours on surfaces, it is mandatory to disinfect what the patient, dentist, and staff have been in contact with. In fact, the virus can remain on metal, plastic, or glass surfaces for up to 9 days. However, it is inactivated by specific substances, such as 70% hydrogen alcohol solutions, 0.5% hydrogen peroxide, or 0.1% sodium hypochlorite used for 1 min. Alternatively you can use sanitizers that contain ozone. Disinfectants with 0.2% chlorhexidine and 0.2% benzyl chloride were found to be less effective [35]. From our study, it emerged that in 96.1% of cases, the operating area was thoroughly sanitized with a dedicated product [24,33,34,35].

In the survey, dentists declared that in 20% of cases, the dental staff were infected, a result which could be related to the non-observance of clinical recommendations during dental visits or to the lack of preventive triage procedures [36].

Another important measure implemented in dental clinical practice to prevent SARS-CoV-2 infection was antisepsis of the mouth before dental care. In fact, it has been described in the literature that the oral mucosa, in particular the tongue, is rich in ACE2 receptors, which allows the entry of COVID-19 into human cells, and that saliva is a potential reservoir for the transmission of COVID-19 [37]. Therefore, although we have no clinical evidence that mouthwashes can prevent transmission of SARS-CoV-2, the use of preoperative antimicrobial mouthwashes with chlorhexidine gluconate (CHX), cetylpyridinium chloride (CPC), and povidone iodine (PVP-I) is recommended. Hydrogen peroxide (H_2_O_2_) to reduce the number of microorganisms in aerosols and drops during oral procedures is also recommended [38]. Mouthwashes with 0.12% chlorhexidine antiseptic are the most common products used [39].

Another prevention measure is the COVID-19 vaccine [40]. In our study, 99.3% of dentists were administered a COVID-19 vaccination and 80% were administered the COVID-19 booster dose vaccination. despite this, 10% of the vaccinated dentists reported a SARS-CoV-2 infection.

### 4.3. Perception of Costs Incurred for all Protective Devices and Measures

All preventive measures implemented in dental practices had costs. About 50% of the dentists interviewed considered the increase in costs adequate, while 47.9% considered it excessive. None of them rated it negligible, and, in the future, this may lead to a reduction of the preventive measures that could be harmful to both dentists and patients. In fact, for 57.9% of dentists interviewed, the COVID-19 vaccination was enough to reduce the preventive measures taken during the pandemic. Nevertheless, vaccine efficacy or effectiveness against infection and symptomatic disease decreased over time [41].

On the other hand, the implementation of the guidelines turned into an unavoidable increase in costs for dentists who have had to raise the prices of dental services, which has therefore had an impact on patients’ budgets [42,43]. After all, PPE and preventive measures have a relatively low cost, especially when compared to the cost of infecting the operator, his staff, or a patient. Nevertheless, it remains important to consider that new SARS-CoV-2 variants or other virus outbreaks, such as Monkeypox, raise the need to maintain preventive measures, that should be constantly implemented and adjusted, according to newly emerging pathogens and those reappearing in a new guise, as also underlined by Khanagar et al. [44,45,46].

The results of this study are also In agreement with another study by Paolone et al., which highlighted the changes and impact of COVID-19 on dental professionals one year from the outbreak compared to the pre-pandemic period [47].

The limitation of the study is that it is a survey-based study, so the participants self-reported the information. However, it also presents some strengths, such as the high number of participants and the good distribution of the respondent’s characteristics.

## 5. Conclusions

The majority of Italian dentists implemented the use of PPE and SARS-CoV-2 infection prevention measures in their practice and maintained them even after the critical breakthrough in 2020.

The most commonly used PPE are facemasks, but most respondents stated that they change it every 8 h or more, thus increasing the risk of being infected.

All respondents continued to triage patients at the entrance of dental practice, 96.1% of them continued to sanitize the operating area after every appointment, and 99.3% of them had had the SARS-CoV-2 vaccine.

Almost half of the Italian dentists considered the cost of maintaining preventive measures and PPE excessive.

## Figures and Tables

**Table 1:** Personal data and professional characteristics of dentists.

		Frequency	Percentage
Gender	M	182	65%
	F	98	35%
	Other	0	0%
Age (year old)	<35	68	24.3%
	35–45	112	40%
	45–55	42	15%
	55–65	36	12.9%
	>65	22	7.8%
SARS-CoV-2 infection	Yes	224	80%
	No	56	20%
COVID-19 vaccination	Yes	278	99.3%
	No	2	0.7%
COVID-19 booster dose vaccination	Yes	224	80%
	No	56	20%
Place of work	Italy	275	98.2%
	Abroad	5	1.8%
	Both	0	0%
Main practice	Oral surgery	62	22.1%
	Endodontics	34	12.1%
	Orthodontics	62	22.1%
	Pediatric dentistry	22	7.9%
	Periodontology	14	5%
	Prosthetic/Implantology	50	17.9%
	Others	36	12.9%

**Table 2:** Measures implemented and devices used daily in dental clinical practice to prevent SARS-CoV-2 infection.

		Frequency	Percentage
PPE implementation in clinical practice	Yes	266	95%
	No	14	5%
Detection of patients’ temperature on arrival	Yes	280	100%
	No	0	0%
During triage procedures recorded temperature ≥37.5 °C	Yes	92	32.9%
	No	188	67.1%
In case of temperature ≥37.5 °C rescheduled a new appointment (deferable treatments)	Yes	92	100%
	No	0	0%
COVID patient visited or treated for dental emergencies	Yes	30	10.7%
	No	250	89.3%
Are you aware of any cases of clinician or staff infection that allegedly occurred within dental office/clinic?	Yes	56	20%
	No	224	80%
Mask routinely worn in the study (non-operative phase)	FFP2	154	55%
	FFP3	10	3.6%
	FFP1 or surgical	41	14.6%
	FFP2 + surgical	55	19.6%
	FFP3 + surgical	10	3.6%
	None	10	3.6%
Mask worn in the study in operative phase	FFP2	168	60%
	FFP3	10	3.6%
	FFP1 or surgical	10	3.6%
	FFP2 + surgical	84	30%
	FFP3 + surgical	8	2.8%
	None	0	0%
Average mask replacement frequency	After any clinical procedure	42	15%
	Every hour	14	5%
	Every 2 h	3	1%
	Every 4 h	56	20%
	Every 8 h	84	30%
	Once a day	81	29%
Hand sanitization before any procedure (hydroalcoholic solution/water and soap)	Yes	258	92.1%
	No	22	7.9%
Hand sanitization after any Procedure (hydroalcoholic solution/water and soap)	Yes	269	96.1%
	No	11	3.9
Visor use	Yes	224	80%
	No	56	20%
Use of goggles under the visor	Yes	146	65.2%
	No	78	34.8%
Use of disposable gowns/suits	Yes	154	55%
	No	126	45%
Average frequency of replacement of disposable gowns/suits	After any clinical procedure	37	24%
	Twice a day	35	22.7%
	At the end of the day	60	39%
	Other	22	14.3%
Use of scrub hat/disposable headgear	Yes	126	45%
	No	154	55%
Scrub hat/disposable headgear replacements between patients	Yes	92	73%
	No	34	27%
Patients wear PPE to access the operating area	Yes	182	65%
	No	98	35%
PPE that patients wear before entering the operating area	Shoe cover	150	82.4%
	Disposable gowns/suits	54	29.7%
	Disposable headgear	70	38.5%
Operating room ventilated for at least 15 min between patients	Yes	190	67.9%
	No	90	32.1%
The operating area is thoroughly sanitized with dedicated products between patients	Yes	269	96.1%
	No	11	3.9%

**Table 3:** Costs incurred for all protective devices and measures and their help in preventing infection.

		Frequency	Percentage
The cost incurred for the purchase of PPE	Negligible	0	0%
	Adequate	146	52.1%
	Expensive	134	47.9%
Do you think the measures you adopted were helpful in preventing COVID-19 infection?	Yes	269	96.1%
	No	11	3.9%
Did the adopted protocols become lighter after the anti-COVID-19 vaccination?	Yes	118	42.1%
	No	162	57.9%

## Data Availability

Data are available upon request to the contact author.
